# The Importance of PTH for Heart Failure. Comment on Thiele et al. The Role of Vitamin D_3_ as an Independent Predicting Marker for One-Year Mortality in Patients with Acute Heart Failure. *J. Clin. Med.* 2022, *11*, 2733

**DOI:** 10.3390/jcm11206038

**Published:** 2022-10-13

**Authors:** William B. Grant, Edward B. Jude

**Affiliations:** 1 Sunlight, Nutrition and Health Research Center, P.O. Box 641603, San Francisco, CA 94164-1603, USA; 2 Division of Diabetes, Endocrinology & Gastroenterology, School of Medical Sciences, Faculty of Biology, Medicine and Health, The University of Manchester, Oxford Road, Manchester M13 9PL, UK; 3 Faculty of Science and Engineering, Manchester Metropolitan University, John Dalton Buliding, Oxford Road, Manchester M1 5GD, UK

The communication by Thiele et al. reported that there were no significant differences in serum 25-hydroxyvitamin D (25(OH)D) for the 22 of 118 acute heart failure (HF) patients, 71% with de novo HF and 29% with chronic HF, who died within 12 months after admission to the intermediate care unit of a maximum care hospital in Germany compared to those who survived [1]. The authors noted that this outcome differed from what was expected from the Seattle Heart Failure Model [2].

However, the authors did not consider other factors that may be important, such as the role of the parathyroid hormone (PTH) in HF. Several articles have reported that PTH concentrations but not 25(OH)D concentrations predict worse survival with chronic HF. One study that included 2312 participants free of cardiovascular disease followed up for a median of 14 years found that serum PTH concentrations ≥ 65 pg/mL were associated with an adjusted hazard ratio (aHR) for heart failure of 1.47 (95% confidence interval (CI) 1.19–1.80, *p* < 0.001) as well as cardiovascular death (1.30 (95% confidence interval (CI), 1.05–1.61, *p* = 0.03), but not all-cause mortality [3]. In that study, the serum 25(OH)D concentration was not significantly correlated with incident HF, but 25(OH)D < 15 ng/mL was associated with a 29% (95% CI: 5–55%) greater risk of mortality.

Another study that followed 3731 men in the UK for 13 years found that elevated PTH (≥55.6 pg/mL; top quarter) was associated with a significantly higher risk of incident HF after adjustments for lifestyle characteristics, diabetes mellitus, blood lipids, blood pressure, lung function, heart rate, renal dysfunction, atrial fibrillation, forced expiratory volume in 1 s, and C-reactive protein (HR = 1.66 (95% CI, 1.30–2.13)) [4].

Some have reported that both PTH and 25(OH)D were independently or together associated with chronic HF survival. In a 3.5-year follow-up study of 148 HF outpatients with a mean age of 68 years in Denmark, both vitamin D and PTH were also significantly associated with all-cause mortality, with aHR of 1.9 (95% CI, 1.1–3.4) for 25(OH)D < 30 ng/mL and 2.0 (95% CI, 1.0–3.8) for PTH > 4.45 pM (42.0 pg/mL), respectively [5].

Previous studies did find 25(OH)D concentrations that were inversely correlated with chronic HF survival.

In a large study (*n* = 49,825, 3009 with HF) with a HF group (mean age of 76 ± 11 years) and control group (mean age of 64 ± 12 years), the patients with HF had a lower median 25(OH)D level compared to the control group (36.9 nmol/L vs. 40.7 nmol/L; *p* < 0.00001)) [6], whereas PTH was significantly higher in the HF group vs. the control group (198 (84–226) vs. 77 (52–118) pg/mL, *p* < 0.00001), but mortality data were not mentioned.

A study in Turkey followed 302 patients with HF for about four years [7]. The aHR for a low 25(OH)D concentration was 0.93 (95%CI, 0.89–0.97). Survival rates as a function of the 25(OH)D concentration were 74% for 25(OH)D < 13.3 ng/mL, 88% for 25(OH)D between 13.3 and 20.3 ng/mL, and 92% for 25(OH)D > 20.3 ng/mL. Mean PTH concentrations were 96 ng/L for 25(OH)D < 13.3 ng/mL, 66 ng/L for 25(OH)D between 13.3 and 20.3 ng/mL, and 61 ng/L for 25(OH)D >20.3 ng/mL, and that the r-coefficient for PTH with 25(OH)D was −0.34 (*p* < 0.001). However, no analysis for the effect of PTH on survival was reported.

Aldosterone levels are raised in heart failure, resulting in secondary hyperaldosteronism as a consequence of renin–aldosterone axis activation, leading to salt and fluid retention with worsening of HF, morbidity, and mortality. Recent evidence suggests that aldosterone might functionally interact with the parathyroid hormone, and significant increases in PTH levels have been observed in patients with hyperaldosteronism [8,9,10]. It has also been shown that type 1 PTH receptors are expressed in aldosterone-producing adenomas, which explains why PTH elevation might increase aldosterone secretion.

PTH is increased as a result of (1) the MR (mineralocorticoid receptor)-mediated calciuretic and magnesiuretic effects, with a trend for hypocalcemia and hypomagnesemia; the resulting secondary hyperparathyroidism causes myocardial fibrosis and disturbed bone metabolism. PTH increase is also the result of the direct effects of aldosterone on parathyroid cells via binding to the MR (2). This adverse sequence is interrupted by mineralocorticoid receptor blockade and adrenalectomy. Hyperaldosteronism can also result in deficiency of the klotho gene that mediates FGF23, which regulates calcium and phosphate homeostasis. FGF23 has been shown to increase urinary excretion of phosphate and to suppress intestinal phosphate absorption but renal 1α-hydroxylase expression, a kidney enzyme responsible for 1,25(OH)_2_D production. Soluble klotho directly regulates phosphorous excretion in the kidney and participates in systemic mineral homeostasis by regulating 1 α-hydroxylase activity and results in vascular calcification, which can be mitigated by spironolactone treatment. In view of the documented reciprocal interaction between aldosterone and PTH as well as the potentially ensuing target organ damage, studies are needed to evaluate diagnostic and therapeutic strategies to address this increasingly recognized pathophysiological phenomenon [8].

The reason for the discordance between the various studies may be that PTH increases with age for any 25(OH)D concentration [11]. As shown in Figure 2A of that article, for 25(OH)D concentrations near 6 ng/mL, PTH is near 50 pg/mL for ages < 20 years, 56 pg/mL for 20–40 years, 71 pg/mL for 40–60 years, and 83 pg/mL for those over the age of 60 years.

PTH > 75 pg/mL was found to be an important risk factor for CVD mortality in a study in Utah [12]. An analysis of seasonal changes in mortality rates noted that increases in PTH associated with reduced 25(OH)D concentrations for the elderly in winter could help explain higher CVD mortality rates in winter [13].

Thus, we suggest that the authors of Ref. [1] reanalyze the results of their study in terms of PTH rather than only 25(OH)D concentrations. PTH (parathormone) concentrations are reported in Table 1 (36.50 *±* 29.64 pg/mL). It would be interesting to see if there was any association between PTH levels and acute HF and mortality.

## References

[1] Thiele K., Cornelissen A., Florescu R., Kneizeh K., Brandenburg V.M., Witte K., Marx N., Schuh A., Stohr R. (2022). The Role of Vitamin D_3_ as an Independent Predicting Marker for One-Year Mortality in Patients with Acute Heart Failure. J. Clin. Med..

[2] Levy W.C., Mozaffarian D., Linker D.T., Sutradhar S.C., Anker S.D., Cropp A.B., Anand I., Maggioni A., Burton P., Sullivan M.D. (2006). The Seattle Heart Failure Model: Prediction of survival in heart failure. Circulation.

[3] Kestenbaum B., Katz R., de Boer I., Hoofnagle A., Sarnak M.J., Shlipak M.G., Jenny N.S., Siscovick D.S. (2011). Vitamin D, parathyroid hormone, and cardiovascular events among older adults. J. Am. Coll. Cardiol..

[4] Wannamethee S.G., Welsh P., Papacosta O., Lennon L., Whincup P.H., Sattar N. (2014). Elevated parathyroid hormone, but not vitamin D deficiency, is associated with increased risk of heart failure in older men with and without cardiovascular disease. Circ. Heart Fail..

[5] Schierbeck L.L., Jensen T.S., Bang U., Jensen G., Kober L., Jensen J.E. (2011). Parathyroid hormone and vitamin D--markers for cardiovascular and all cause mortality in heart failure. Eur. J. Heart Fail..

[6] Gotsman I., Shauer A., Zwas D.R., Hellman Y., Keren A., Lotan C., Admon D. (2012). Vitamin D deficiency is a predictor of reduced survival in patients with heart failure; vitamin D supplementation improves outcome. Eur. J. Heart Fail..

[7] Yilmaz Oztekin G.M., Genc A., Arslan S. (2021). Vitamin D Deficiency Is a Predictor of Mortality in Elderly with Chronic Heart Failure. Acta Endocrinol..

[8] Tomaschitz A., Ritz E., Pieske B., Rus-Machan J., Kienreich K., Verheyen N., Gaksch M., Grubler M., Fahrleitner-Pammer A., Mrak P. (2014). Aldosterone and parathyroid hormone interactions as mediators of metabolic and cardiovascular disease. Metabolism.

[9] Asbach E., Bekeran M., Reincke M. (2015). Parathyroid Gland Function in Primary Aldosteronism. Horm. Metab. Res..

[10] Vaidya A., Brown J.M., Williams J.S. (2015). The renin-angiotensin-aldosterone system and calcium-regulatory hormones. J. Hum. Hypertens..

[11] Valcour A., Blocki F., Hawkins D.M., Rao S.D. (2012). Effects of age and serum 25-OH-vitamin D on serum parathyroid hormone levels. J. Clin. Endocrinol. Metab..

[12] Anderson J.L., Vanwoerkom R.C., Horne B.D., Bair T.L., May H.T., Lappe D.L., Muhlestein J.B. (2011). Parathyroid hormone, vitamin D, renal dysfunction, and cardiovascular disease: Dependent or independent risk factors?. Am. Heart J..

[13] Grant W.B., Boucher B.J. (2022). An Exploration of How Solar Radiation Affects the Seasonal Variation of Human Mortality Rates and the Seasonal Variation in Some Other Common Disorders. Nutrients.

